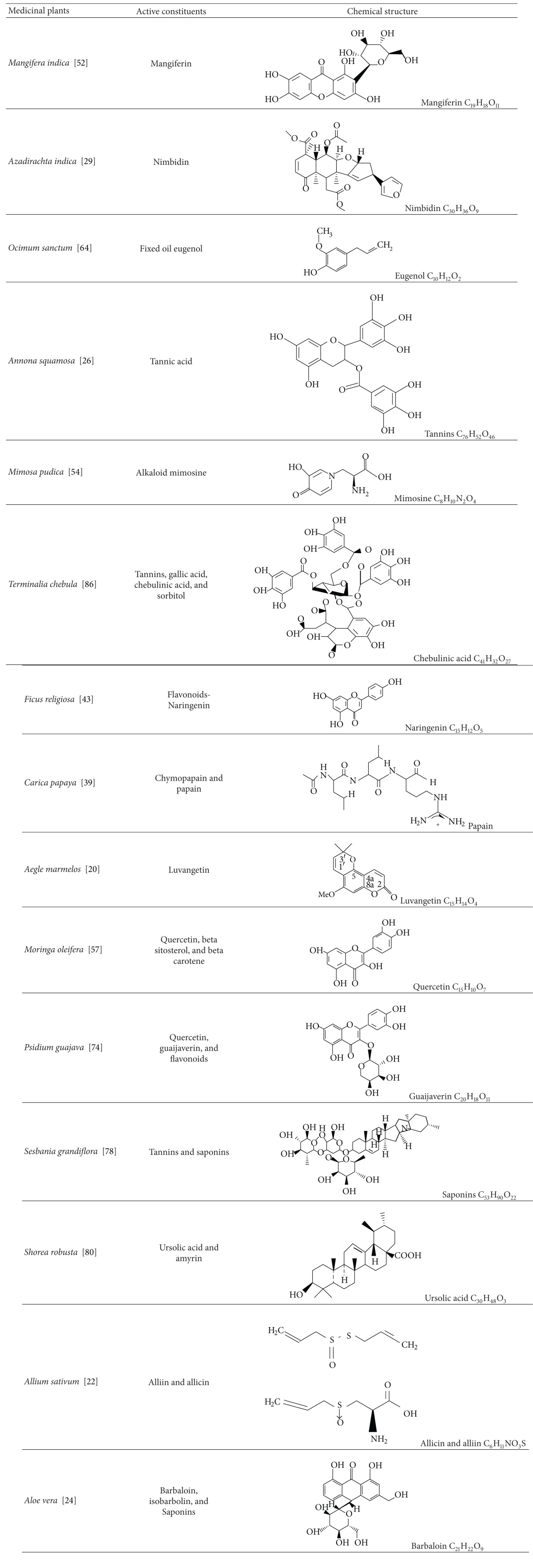# A Review on Antiulcer Activity of Few Indian Medicinal Plants

**DOI:** 10.1155/2014/519590

**Published:** 2014-05-25

**Authors:** G. Vimala, F. Gricilda Shoba

**Affiliations:** PG & Research Department of Zoology, Voorhees College, Vellore, Tamilnadu 632001, India

## Abstract

Ulcer is a common gastrointestinal disorder which is seen among many people. It is basically an inflamed break in the skin or the mucus membrane lining the alimentary tract. Ulceration occurs when there is a disturbance of the normal equilibrium caused by either enhanced aggression or diminished mucosal resistance. It may be due to the regular usage of drugs, irregular food habits, stress, and so forth. Peptic ulcers are a broad term that includes ulcers of digestive tract in the stomach or the duodenum. The formation of peptic ulcers depends on the presence of acid and peptic activity in gastric juice plus a breakdown in mucosal defenses. A number of synthetic drugs are available to treat ulcers. But these drugs are expensive and are likely to produce more side effects when compared to herbal medicines. The literature revealed that many medicinal plants and polyherbal formulations are used for the treatment of ulcer by various ayurvedic doctors and traditional medicinal practitioners. The ideal aims of treatment of peptic ulcer disease are to relieve pain, heal the ulcer, and delay ulcer recurrence. In this review attempts have been made to know about some medicinal plants which may be used in ayurvedic as well as modern science for the treatment or prevention of peptic ulcer.

## 1. Introduction

Ulcers are an open sore of the skin or mucus membrane characterized by sloughing of inflamed dead tissue [1]. Ulcers are lesions on the surface of the skin or a mucous membrane characterized by a superficial loss of tissue. Ulcers are most common on the skin of the lower extremities and in the gastrointestinal tract, although they may be encountered at almost any site. There are many types of ulcer such as mouth ulcer, esophagus ulcer, peptic ulcer, and genital ulcer. Of these peptic ulcer is seen among many people. The peptic ulcers are erosion of lining of stomach or the duodenum [2]. The two most common types of peptic ulcer are called “gastric ulcer” and “duodenal ulcer.” The name refers to the site of ulceration. A person may have both gastric and duodenal ulcers at the same time. Gastric ulcers are located in the stomach, characterized by pain; ulcers are common in older age group. Eating may increase pain rather than relieve pain. Other symptoms may include nausea, vomiting, and weight loss. Although patients with gastric ulcers have normal or diminished acid production, yet ulcers may occur even in complete absence of acid [3]. Duodenal ulcers are found at the beginning of small intestine and are characterized by severe pain with burning sensation in upper abdomen that awakens patients from sleep. Generally, pain occurs when the stomach is empty and relieves after eating. A duodenal ulcer is more common in younger individuals and predominantly affects males. In the duodenum, ulcers may appear on both the anterior and posterior walls [4]. In some cases, peptic ulcer can be life threatening with symptoms like bloody stool, severe abdominal pain, and cramps along with vomiting blood [5].

The pathophysiology of peptic ulcer disease involves an imbalance between offensive (acid, pepsin, and* Helicobacter pylori*) and defensive factors (mucin, prostaglandin, bicarbonate, nitric oxide, and growth factors) [6]. Peptic ulcers are once believed to be caused by spicy food and stress; these have been found merely to be aggravating factors and the real causes have been found by research to include bacterial infection (*Helicobacter pylori*) or reaction to various medications, particularly NSAIDS (nonsteroidal anti-inflammatory drugs) [7].* Helicobacter pylori*, NSAIDS drugs, emotional stress, alcohol abuse, and smoking are the principal etiological factors associated with peptic ulcer [8]. The Gram-negative bacterium* Helicobacter pylori* remains present between the mucous layer and the gastric epithelium and is strategically designed to live within the aggressive environment of the stomach. Initially,* Helicobacter pylori* resides in the antrum but over time migrates toward the more proximal segments of the stomach [9].

Peptic ulcer is one of the world's major gastrointestinal disorders and affecting 10% of the world population [10]. About 19 out of 20 peptic ulcers are duodenal. An estimated 15000 deaths occur each year as a consequence of peptic ulcer. Annual incidence estimates of peptic ulcer hemorrhage and perforation were 19.4–57 and 3.8–14 per 100,000 individuals, respectively. The average 7-day recurrence of hemorrhage was 13.9% and the average long-term recurrence of perforation was 12.2% [11]. In the Indian pharmaceutical industry, antacids and antiulcer drugs share 6.2 billion rupees and occupy 4.3% of the market share [6].

In this modern era also 75–80% of the world populations still use herbal medicine mainly in developing countries, for primary health care because of better cultural acceptability, better compatibility with the human body, and lesser side effects [12]. Histological studies revealed that these medicinal plants did not show any acute toxicity. Preliminary photochemical screening of this medicinal plant identified the presence of important secondary metabolites like flavonoids and tannins which are the active principles of antiulcer activity [13].

Present study was conducted to review medicinal plants considered as gastroprotective and healing agents on ulcers in ayurvedic resources and beside that to gather evidence for their effectiveness and biological mechanisms in modern investigation.

In order to achieve this aim, Indian ayurvedic book Meteria Medica and electronic databases including science direct, pubmed, scopus, and google scholar were explored for each of the medicinal plants for peptic ulcers and all retrieved articles were evaluated to achieve any in vitro, in vivo, or clinical evidence for their efficacy and possible mechanisms. The retrieved studies either demonstrate obviously effectiveness of these herbs or indirectly their efficacy on the involved mechanisms in the treatment of peptic ulcers.

Meteria Medica provides lots of information about ethno medicinal herbs, which are valuable as antiulcer agents and their use experimentally was evaluated and proved by many researchers for its antiulcer activity. Following compiled data suggested that medicinal plant those are evidently reported for its antiulcer activity.

## 2. Findings and Discussion

### 2.1. *Acacia arabica*

*Acacia arabica* (family* Mimosaceae*), is common all over India in dry and sandy localities. It is commonly known as “babul tree” and locally called as “karuvelam.” Chemical constituents reported in this plant are gum containing arabic acid combined with calcium, magnesium, and potassium and also small quantity of malic acid, sugar, moisture 14%, and ash 3-4%. Bark contains a large quantity of tannin; pods contain about 22.44% tannin [14].

#### 2.1.1. Antiulcer Activity

*In Ayurvedic*. As gargle it is useful as wash in haemorrhagic ulcer and wounds. Bruised tender leaves formed into a poultice and applied to ulcers act as stimulant and astringent [14].

*In Recent Studies*.* Acacia senegal* gum protected against cold restraint stress-induced gastric ulcer in rats [15]. Aqueous extract of* A. arabica* gum showed protection against meloxicam-induced intestinal damage and attenuated intestinal enzymes activity [16].

*Active Constituents*. Phenolic compounds, tannins, and flavonoids are considered.

### 2.2. *Adansonia digitata*

*Adansonia digitata* belonging to the family* Malvaceae* is commonly known as “boabab or monkey-bread tree of Africa.” It is locally known as “paparapuli.” It is one of the largest and long-lived trees in the world, met with chiefly in Bombay, Gujarat, and Coromandal Coast and Ceylon. Chemical constituents in this plant are Pulp that contains phobaphenes, mucilage and gum, glucose, tartrate and acetate of potash, and other salts. A leaf contains wax, glucose, salts, gum, and albuminoids. Bark contains wax, soluble and insoluble tannin, acid gum, albuminous carbonate and chloride of sodium and potassium, and a glucoside adansonin [17].

#### 2.2.1. Antiulcer Activity

*In Ayurvedic*. Fresh juice of the leaves mixed with powdered ginger together with the expressed juice of the fresh root of* Salvadora indica* is applied with considerable benefit to indolent syphilitic ulcer. Leaves are used as fomentations and poultices for irritable inflammatory ulcers [17].

### 2.3. *Aegle marmelos*

*Aegle marmelos* which is commonly known as a “bael tree” belonging to the family* Rutaceae* is the plant that chiefly grows on throughout India. It is locally called as “vilvam.” Chemical constituents in this plant are flavonoids, tannins, and saponins [18].

#### 2.3.1. Antiulcer Activity

*In Folk Medicine*. The fruit of* A. marmelos* is traditionally used for the treatment of ulcer among the kani tribes in Kanyakumari district, Tamil Nadu, India [18].

*In Recent Studies*. Ulcers are induced by aspirin plus pylorus ligated gastric ulceration in rats and aqueous extract of leaves is to be administered orally for 21 days, daily dose of 1 gm/kg. The result indicated a significant reduction in the ulcer lesion count compared to control [19].

*Active Constituents*. Luvangetin, a pyranocoumarin isolated from the seeds [20], is considered.

### 2.4. *Allium sativum*

*Allium sativum* belonging to the family* Liliaceae* is commonly known as “garlic” and locally called as “vellapundu.” It is cultivated all over India. Chemical constituents in this plant arean acrid volatile oil which is the active principle, starch, mucilage, albumen, and sugar. Seeds yield aromatic oil. The juice, more particularly its oil constituents, is rich in organically bound sulphur, iodine, and salicylic acid combinations, apart from important nutrient and complementary substances containing vitamins [21].

#### 2.4.1. Antiulcer Activity

*In Ayurvedic*. Mustard or coconut oil in which garlic has been fried is an excellent application for maggots infesting ulcers, ulcerated surfaces, and wounds. Garlic juice mixed with 3 or 4 parts of ordinary or distilled water has been used as a lotion for washing wounds and foul ulcers [21].

*In Recent Studies*. The extract of* A. sativum* bulb juice was administered at the doses of 250 and 500 mg/kg orally in rats, against cysteamine induced gastric ulcer. The extract significantly increases healing of gastric ulcer and prevents the development of experimentally induced gastric and duodenal ulcers in rats [22].

*Active Constituents*. Volatile oil, alliin, and allicin are considered.

### 2.5. *Aloe vera*

*Aloe vera* belonging to the family* Liliaceae* is commonly known as “aloe gel.” It is locally called “kattalai” which is found all over India. Chemical constituents in this plant are aloin, isobarbaloin, and emodin [23].

#### 2.5.1. Antiulcer Activity

*In Ayurvedic*. Leaves are being used successfully in America in the local treatment of chronic ulcers. First the pain diminishes and after a few weeks the ulcers heal [23].

*In Recent Studies*.* Aloe vera* powder was mixed with gum acacia; the solution was administered orally in rats at dose of 200 mg/kg against indomethacin induced gastric ulcer. The extract showed significant antiulcer activity comparable to control [24].

*Active Constituents*. Barbalin, isobarbolin, and saponins are considered.

### 2.6. *Annona squamosa*

*Annona squamosa* (*Annonaceae*) is commonly known as “custard apple.” It is cultivated in gardens all over India which is locally called as “sitapalam.” Chemical constituents in this plant are alkaloids, flavonoids, saponins, and tannins. Seeds yield oil and resin; seeds, leaves, and immature fruit contain an acrid principle [25].

#### 2.6.1. Antiulcer Activity

*In Ayurvedic*. Leaves made into a paste without adding water are applied to unhealthy ulcers [25].

*In Recent Studies*. The aqueous leaf extract protected against pylorus ligation and ethanol induced gastric ulcer in rats [26].

*Active Constituents*. Tannic acid is considered.

### 2.7. *Azadirachta indica*

*Azadirachta indica* (family* Meliaceae*) is indigenous to and cultivated nearly all over India and in Bengal. It is commonly known as “neem” and locally called “vembu.” Chemical constituents reported in this plant are nimbidin, phenolic compounds, saponin, and flavonoids. It contains a bitter alkaloid named Margosine. Seeds contain about 10–31% of a yellow bitter fixed oil. The oil contains free and volatile fatty acids. The volatile fatty acids probably consist of a mixture of stearic and oleic acids with a small amount of lauric acid [27].

#### 2.7.1. Antiulcer Activity

*In Ayurvedic*. A poultice of leaves mixed with sesamum seeds is very useful in unhealthy ulcerations [27].

*In Recent Studies*.* Azadirachta indica* leaf extract protected against pylorus ligation and cold restraint stress induced gastric ulcer in rats [28].

*Active Constituents*. Stearic and palmitic acid isolated from the nimbidin fraction of neem seeds oil is considered [29].

### 2.8. *Balsamodendron mukul*

*Balsamodendron mukul* (*Burseraceae*) is commonly known as “gum-gugul.” It is grown on the Sind, Rajputana, Eastern Bengal, Berars, Assam, Khandesh, and Mysore which is locally called “gukkulu.” Chemical constituents in this plant are volatile oil, gum-resin, and bitter principles [30].

#### 2.8.1. Antiulcer Activity

*In Ayurvedic*. Guggul gum is mixed with lime juice or coconut oil; it is applied as a plaster or in the form of a lotion in indolent ulcers. Gum obtained from other species,* B. pubescens* found in Sind, Karachi, and Baluchistan, is used as ointment in bad ulcers such as Delhi sores, combined with sulphur, catechu, and borax [30].

### 2.9. *Bauhinia variegate*

*Bauhinia variegate* (family* Caesalpiniaceae*) is indigenous to and grow on the Sub-Himalayan tract and the forests of India and Burma. It is commonly known as “orchid tree” and locally called “shemmandarai.” Chemical constituents reported in this plant are quercetin, rutin, apigenin, and apigenin 7-0-glucoside. Bark contains tannin (tannic acid), glucose, and a brownish gum [31].

#### 2.9.1. Antiulcer Activity

*In Ayurvedic*. Decoction of the bark is a useful wash in ulcers. A preparation known as kanchanara guggula made of the following ingredients is useful in ulcers: take the bark of* Bauhinia variegate* (10 parts), 3 myrobalans, ginger, black-pepper, long-pepper, bark of Crataeva nurvala, cardamoms, cinnamon, and Tejpatra leaves, each one part. Powder them all and add guggula (15 parts) to make a pill mass. This is given in doses of half a tola every morning with a decoction of* Sphaeranthus mollis* or of Triphala or of catechu [31].

*In Recent Studies*. The ethanolic and aqueous extract of root of* B. variegate* was administered at the doses of 200 and 400 mg/kg orally, in rats against pylorus ligation, ethanol, and aspirin induced gastric ulcer. The extract significantly inhibited gastric mucosal damage and reduced the basal gastric acid secretion [32].

*Active Constituents*. Flavonoids are considered.

### 2.10. *Berberis aristata*

*Berberis aristata* (family* Berberidaceae*) is grown on the Nilgiris and all over the temperate Himalayas, from Bhutan to Kunawer. It is commonly known as “Indian or Nepal barberry” and locally called “kasturimanjal.” Chemical constituents reported in this plant are roots and wood which are rich in a yellow alkaloid “berberine” bitter substance, which dissolves in acids and forms salts of the alkaloid; root contains two more alkaloids [33].

#### 2.10.1. Antiulcer Activity

*In Ayurvedic*. Crude extracts known as rasaut (in Hindi) are prepared from the root; bark mixed with honey is useful application to ulcerations of the skin [33].

### 2.11. *Beta vulgaris*

*Beta vulgaris* (*Chenopodiaceae*) is commonly known as “beetroot.” It is native of the sea-coasts of tree Mediterranean, now extensively cultivated in Europe and America, and is known as sugar-beet. It is also cultivated in gardens in many parts of India for the sake of its flesh roots and leaves. There are two kinds: white and red. Chemical constituents in this plant are an active principle “betin” [34].

#### 2.11.1. Antiulcer Activity

*In Ayurvedic*. A decoction of the root with a little vinegar added is excellent for all kinds of ulcers and running sores [34].

### 2.12. *Careya arborea*

*Careya arborea* (*Myrtaceae*) is commonly known as “slow match tree.” It is locally called “pailacputatammi.” It is frequent in Sub-Himalayan tract. Chemical constituents in this plant are thick red bark containing tannin 8%. Liber contains calcium oxalate in large simple crystals [35].

#### 2.12.1. Antiulcer Activity

*In Ayurvedic*. Leaves made into a pulp and used as poultice 3 to 4 times a day rapidly heal obstinate ulcers [35].

*In Recent Studies*. The ethanol stem bark extract of* C. arborea* was administered at the doses of 300 and 600 mg/kg orally in rats for 5 days against ethanol, cold restraint stress, and pylorus ligation induced ulcer models. The extract significantly increases healing of gastric ulcer as compared to control [36].

*Active Constituents*. Tannins and saponins are considered.

### 2.13. *Carica papaya*

*Carica papaya* (*Caricaceae*) is commonly known as “papaya.” It is locally called “papali-pazham.” It grows in all tropical countries and many subtropical regions of the world. Chemical constituents in this plant are Papain, chymopapain, pectin, carposide, carpaine, carotenoids, and antheraxanthin [37].

#### 2.13.1. Antiulcer Activity

*In Folk Medicine*. It is largely used in tropical folk medicines. The ripe fruit is edible and unripe can be eaten cooked for indolent ulcer. The unripe fruit can be cooked as parts of salads, jellies, and stews while the ripe fruits are usually eaten raw without the skin or seed. Intake of the unripe fruit of the plant has been linked with an antiulcer effect [37].

*In Recent Studies*. The aqueous seed extract of* C. papaya* was administered at the doses of 50 and 100 mg/kg orally, in rats against ethanol induced gastric ulcer. The extract protected the gastric mucosa against ethanol effect.* C. papaya* extract significantly reduced the gastric juice volume and gastric acidity [38].

*Active Constituents*. Chymopapain and papain are widely known as being useful for digestive disorders and disturbances of the gastrointestinal tract [39].

### 2.14. *Euphorbia neriifolia*

*Euphorbia neriifolia* (*Eurphorbiaceae*) is commonly known as “common milk hedge.” It is locally called “ilaikkalli.” This leafless shrub is found in Central India and cultivated in Bengal. Chemical constituents in this plant are Euphorbon, resin, gum, caoutchouc, malate of calcium, and so forth [40].

#### 2.14.1. Antiulcer Activity

*In Ayurvedic*. Plant juice is largely used with clarified or fresh butter as an application to unhealthy ulcers and scabies [40].

### 2.15. *Ficus religiosa*

*Ficus religiosa* (*Urticaceae*) is commonly known as “sacred fig.” It is locally called “arasha-maram.” This sacred peepul is a large tree round wild and cultivated all over India by the Hindus. Chemical constituents in this plant are bark containing tannin, caoutchouc (cochtone), and wax [41].

#### 2.15.1. Antiulcer Activity

*In Ayurvedic*. Bark is useful in ulcers in infusion or decoction (simple kashayam) with a little honey [41].

*In Recent Studies*. The hydro alcoholic extract leaves of* F. religiosa* were studied at two dose levels (250 and 500 mg/kg, oral) in rats against absolute ethanol, aspirin, and pylorus ligation induced gastric ulcer. The extract significantly decreases the ulcer index value when compared to control [42].

*Active Constituents*. Bioactive compounds like flavonoids, saponins, and tannins are considered [43].

### 2.16. *Galega purpurea*

*Galega purpurea* (*Papilionaceae*) is commonly known as “purple tephrosia.” It is locally called “kolluk-kay-welai.” It is found throughout India, especially in Southern India. It grows on hard stony ground too difficult to be rooted. Chemical constituents in this plant are yields gum, a trace of albumen and colouring matter, ash containing a trace of manganese, brown resin and chlorophyll and a principle allied to quercetin or querritrin, and glucoside rutin [44].

#### 2.16.1. Antiulcer Activity

*In Ayurvedic*. Root powdered and mixed with honey is applied to ulcers [44].

### 2.17. *Hibiscus rosa sinensis*

*Hibiscus rosa sinensis* (*Malvaceae*) is commonly known as “changing rose.” It is locally called “chembaruthi.” It is native to China and grown widely as an ornamental plant through India. Chemical constituents in this plant are flavonoids, anthocyanins, quercetin, cyanidin, kaempferol, and hydrocitric acid [45].

#### 2.17.1. Antiulcer Activity

*In Folk Medicine*. The root of* H. rosa sinensis* is traditionally used for the treatment of ulcer among the kani tribes in Kanyakumari district, Tamil Nadu, India [45].

*In Recent Studies*. The aqueous and alcohol extracts of* H. rosa sinensis* roots possessed significant antiulcer activity in pylorus ligated rats at the doses of 250 and 500 mg/kg. Thus, it has been scientifically proven that these extracts possess enough potential as an antiulcerogenic agent [46].

*Active Constituents*. Flavonoids and quercetin are considered.

### 2.18. *Hydrocotyle asiatica*

*Hydrocotyle asiatica* (*Umbelliferae*) is commonly known as “Indian penny-wort.” It is locally called “vaellarai.” This small weed is common all over India, growing plentifully in moist localities. Chemical constituents in this plant are an oleaginous white crystalline substance vellarin which is the active principle of the leaves, resins and some fatty aromatic body, gum, sugar, tannin, albuminous matter, and salts, mostly alkaline sulphates [47].

#### 2.18.1. Antiulcer Activity

*In Ayurvedic*. For ulcerations, the powder, in 3 to 5 grain doses, may be given thrice daily; at the same time some of the powder may be sprinkled on the ulcers or preferably poultices of the fresh leaves may be applied [47].

### 2.19. *Indigofera tinctoria*

*Indigofera tinctoria* (*Papilionaceae*) is commonly known as “true indigo.” It is locally called “neelum; avari.” This small erect shrub is cultivated extensively in Northern India, especially in Bengal, Bihar, Orissa, Sind, Oudh, Southern India, Madras, and Bombay. Chemical constituents in this plant are Indican (a glucoside), the oxidized form of Luc-indigo, or Indigo-white, what is produced from the fermentation of the fresh green plant [48].

#### 2.19.1. Antiulcer Activity

*In Ayurvedic*. Leaves crushed are used as stimulant poultice or plaster in various skin affections and to cleanse and to heal wounds and ulcers. Powdered indigo is also used for sprinkling on ulcers [48].

### 2.20. *Lawsonia alba*

*Lawsonia alba* (*Lythraceae*) is commonly known as “henna.” It is locally called “maruthoni.” It is common all over India, cultivated chiefly as a hedge and garden plant. Chemical constituents in this plant are leaves that yield a colouring matter (henna dye) 12 to 15% Hanno, tannic acid, a kind of tannin, and an olive green resin soluble in ether and alcohol. Seeds yield oil. There is also glucoside in the plant [49].

#### 2.20.1. Antiulcer Activity

*In Ayurvedic*. An ointment prepared from the leaves is used to cure wounds and ulcers [49].

### 2.21. *Mangifera indica*

*Mangifera indica* (*Anacardiaceae*) is commonly known as “mango tree.” It is locally called “mangaai.” It is cultivated throughout India. Chemical constituents in this plant are alkaloids, sterols, saponins, tannins, and flavonoids [50].

#### 2.21.1. Antiulcer Activity

*In Ayurvedic*. Leaf extracts were dissolved in rice bran oil and given orally for ulcer. Traditionally the plant is reported to have antiulcer activity [50].

*In Recent Studies*. The flower decoction was administered in the doses of 250, 500, and 1000 mg/kg orally, in rats with gastric lesions in dose-dependent manner. Thus, the extract significantly reduced the gastric juice volume and gastric acidity [51].

*Active Constituents*. Mangiferin [52] is considered.

### 2.22. *Mimosa pudica*

*Mimosa pudica* (*Fabaceae*) is commonly known as “touch me not.” It is locally called “thottal sinungee.” It grows in all tropical countries and many subtropical regions of the world. Chemical constituents in this plant are flavonoids, quercitin, naringin, saponins, tannins, gums, and mucilage [53].

#### 2.22.1. Antiulcer Activity

*In Ayurvedic*. Decoction of the fresh leaves and seeds are consumed for intestinal ulcer [53].

*In Recent Studies*. Ethanolic extract of the leaves of* Mimosa pudica* have been reported to possess antiulcer activity in a dose-dependent manner and these leaf extracts may be useful as a natural antioxidant in treatment of ulcer [54].

*Active Constituents*. Alkaloid mimosine is considered.

### 2.23. *Momordica charantia*

*Momordica charantia* (*Cucurbitaceae*) is commonly known as “bitter gourd.” It is locally called as “pavakka-chedi.” This climbing plant is cultivated in gardens everywhere in India, for its fruit. Chemical constituents in this plant are bitter glucoside soluble in water and insoluble in ether, a yellow acid, resin, and ash 6%. Fresh vegetable contains 88.75% moisture, albuminoids 1.62%, soluble carbohydrates 85.41%, woody fiber 1.51%, and ash 8.53% [55].

#### 2.23.1. Antiulcer Activity

*In Ayurvedic*. Whole plant powdered is used for dusting over leprous and other intractable ulcers and in healing wounds; when mixed with cinnamon, long pepper, rice, and chaulmugra oil it forms a good ointment in malignant ulcers [55].

*In Recent Studies*. Alcoholic and aqueous extract of* M. charantia* fruit at the doses of 200 and 400 mg/kg separately are used against pylorus ligation, aspirin, and stress induced ulcer in rats. These extracts showed significant reduction in ulcer index as compared to control [56].

*Active Constituents*. Flavonoids, saponins, and sterols are considered.

### 2.24. *Moringa oleifera*

*Moringa oleifera* (*Moringaceae*) is commonly known as “drum-stick, horse radish tree.” It is locally called “murungai.” It is native to the Western and sub-Himalayan region, India, Pakistan, Asia minor, Africa, and Arabia. Chemical constituents in this plant are alkaloids, flavonoids, saponin, tannins, zeatin, quercetin, kaempferom, and terpenoids [45].

#### 2.24.1. Antiulcer Activity

*In Folk Medicine*. The medicinal value of the different parts of the plant has long been recognized in folklore medicine. The leaf tea treats gastric ulcers by Kani tribals of Pechiparai Hills, Tamil Nadu, India. Flower buds of* M. oleifera* are widely consumed in Pakistan and have been reported to possess antiulcer activity [45].

*In Recent Studies*. The alcoholic leaves extract of* M. oleifera* was administered in the doses of 125, 250, and 500 mg/kg orally, in rats against pylorus ligation, ethanol, cold restraint stress, and aspirin induced gastric ulcer. The extract showed decreases in ulcer and acid pepsin secretion [57]. 

*Active Constituents*. Quercetin, beta sitosterol, and beta carotene are considered.

### 2.25. *Myrica nagi*

*Myrica nagi* (*Myricaceae*) is commonly known as “box myrtle; bay-berry.” It is locally called “marudam-pattai.” It is an evergreen plant of the subtropical Himalayas, Simla District, SyIhet, and southwards to Singapore and found also in the Khasia Mountains and the hills of Burma. This is a very commonly cultivated tree in China and Japan. Chemical constituents in this plant are bark that contains tannin, saccharine matter, and salts. The ground bark yields a colouring principle named “myricotin” [58].

#### 2.25.1. Antiulcer Activity

*In Ayurvedic*. A poultice made by bruising the bark and simmering it in water and stirring in Indian meal till it obtains the proper consistence cures scrofulous ulcers (Tukina). Fruits when boiled yield a kind of wax called myrtle wax which is used as a healing application to ulcers [58].

### 2.26. *Myrtus communis*

*Myrtus communis* (*Myrtaceae*) is commonly known as “myrtle.” It is cultivated in many parts (in gardens) of India. Chemical constituents in this plant are ripe berries that contain an essential volatile oil (oil of Myrtle), resin, tannin, citric acid, malic acid, and sugar. [59].

#### 2.26.1. Antiulcer Activity

*In Ayurvedic*. Powder of leaves is a useful application in wounds and ulcers. The fruit, Myrtle berry, is carminative and given in the form of infusion for internal ulcerations [59].

*In Recent Studies*. A topical formulation of* M. communis* in low doses demonstrated wound healing activity in rat excision wounds [60].* M. communis* fruits protected against gastric ulcer caused by ethanol, indomethacin, and pylorus ligation in rats via suppressing gastric secretion and acidity and enhancing its mucosal barrier [61].

*Active Constituents*. Myrtle (Volatile oil) is considered.

### 2.27. *Ocimum sanctum*

*Ocimum sanctum* (*Lamiaceae*) is commonly known as “holy basil.” It is locally called “tulsi.” It grows throughout India. The name Tulsi means “the incomparable one.” It is one of the sacred herbs for Hindus in the Indian subcontinent. Chemical constituents in this plant are alkaloids, tannins, saponins, flavonoids, and sterols [62].

#### 2.27.1. Antiulcer Activity

*In Ayurvedic*. Indian materia medica describes the use of the plant in a variety of ailments. The fresh leaves are taken as Prasad by millions of Indian for many years. A tea prepared with the leaves of Tulsi is commonly used for intestinal disorders [62].

*In Recent Studies*. The fixed oil of* O. sanctum* was administered in the doses of 1, 2, and 3 mL/kg intraperitoneally, in the rats in which ulcer is induced by aspirin, indomethacin, alcohol, and stress-induced ulceration. It reduces the ulcer index in dose-dependent manner [63].

*Active Constituents*. Fixed oil eugenol [64] is considered.

### 2.28. *Odina wodier*

*Odina wodier* (*Anacardiaceae*) is commonly known as “odiyamaram.” It is cultivated generally in hotter parts of India.Chemical constituents in this plant are barks that contain tannin and ash that contains considerable quantity of potassium carbonate [65].

#### 2.28.1. Antiulcer Activity

*In Ayurvedic*. Fresh juice of the bark is a valuable application to obstinate ulcers. Bark powdered mixed with neem oil is an application for chronic ulcers. Powdered bark is used as a paste for leprous ulcers [65].

### 2.29. *Oryza sativa*

*Oryza sativa* (*Gramineae*) is commonly known as “rice; paddy.” It is locally called “arshi; nellu.” It grows throughout India. This is a principal food crop of India, Ceylon, Burma, China, Japan, and Siam and is spread over the tropical and subtropical regions of both hemispheres. Chemical constituents in this plant are rice that contains more starch than any other starchy grains, but no appreciable fat, a very small quantity of proteins, and a trace of mineral matter [66].

#### 2.29.1. Antiulcer Activity

*In Ayurvedic*. Where there is an irritable or inflammatory state of the stomach, rice gruel or conjee water, as it is commonly called, (Decoction 1 in 40) or thicker liquid made by boiling the rice powder in water, with a pinch of salt and a squeeze of lemon, makes a good drink and without the lime-juice and salt in gastric ulcer. Schnabel in American Journal of Medical Science reports good results from the use of rice-water mixture in the treatment of gastric and duodenal ulcers [66].

*In Recent Studies*. The extract of* O. sativa* bran (rice bran oil) was administered at the dose of 1 mL/day for 4 days against swimming stress induced and pylorus ligation induced ulcer in rats. The extract showed significant reduction in the basal gastric acid secretion [67].

### 2.30. *Peucedanum grande*

*Peucedanum grande* (*Umbelliferae*) is commonly known as “wild carrot.” It is found on the hills of Western India. Chemical constituents in this plant are fruits that contain an essential oil of a light yellow colour [68].

#### 2.30.1. Antiulcer Activity

*In Ayurvedic*. Infusion (1 in 10) of fruit is used in doses of 1*⁄*2 to 1 ounce like that of fennel seeds as carminative, gastric and intestinal disorders, and so forth, [68].

### 2.31. *Phyllanthus niruri*

*Phyllanthus niruri* (*Euphorbiaceae*) is commonly known as “stonebreaker or seed-under-leaf.” It is locally called “kizhkay nelli.” It is common in Central and Southern India, extending to Ceylon. Chemical constituents in this plant are alkaloids, saponins, tannins, flavonoids, carbohydrates, and glycosides [69].

#### 2.31.1. Antiulcer Activity

*In Ayurvedic*. Whole plant pounded with its root and combined with rice water is used as poultice for ulcers [69].

*In Recent Studies*. The metanolic aerial part extract of* P. niruri* was administered at the dose of 400 mg/kg orally in rats and significantly inhibited the development of ulcer induced by indomethacin [70]. 

*Active Constituents*. Alkaloids-4-methoxy-securinine, ellagic acid, beta sitosterol, gallic acid, and hypophyllanthin are considered.

### 2.32. *Pinus longifolia*

*Pinus longifolia* (*Coniferae*) is commonly known as “long-leaved pine.” It is locally called “shirsal.” It is common on the slopes of the Himalayas, North Western Frontier Province from Afghanistan to Kashmir, the Punjab, U.P. to Bhutan, Assam, and Upper and Lower Burma. Chemical constituents: Its sapwood yields on incision an oleoresin from which turpentine is obtained which contains 20% volatile oil of turpentine called pinene with a small quantity of limonene and about 80% of residue which is very largely used under the name of calophony or resin [71].

#### 2.32.1. Antiulcer Activity

*In Ayurvedic*. Wood is useful to cool the burning sensation of the body and as an application in ulcerations. It is the source of the resin usually employed as a stimulating application for ulcers [71].

### 2.33. *Plantago ispagula*

*Plantago ispagula* (*Plantaginaceae*) is commonly known as “spogel seeds.” It is locally called “ishappukolvirai.” This Persian herb is found also in North-West India, the Punjab, and Sind and cultivated to a small extent in Bengal, Mysore, and Coromandel Coast. The genus Plantago comprises about 50 species, of which ten are natives of India. Chemical constituents in this plant are Mucilage, fixed fatty oil, and albuminous matter, in large quantities [72].

#### 2.33.1. Antiulcer Activity

*In Ayurvedic*. The decoction in doses of 2 to 3 drachms, plain or mixed with sugar, is very beneficial in gastritis, gastric, and duodenal ulcers [72].

### 2.34. *Psidium guyava*

*Psidium guyava* (*Myrtaceae*) is commonly known as “guava.” It is locally called “koyya.” This tree is cultivated nearly all over India and is common in Bengal. Chemical constituents in this plant are bark that contains tannin 27.4%, resin, and crystals of calcium oxalate. Leaves contain resin, fat, cellulose, tannin, volatile oil, chlorophyll, and mineral salts [73].

#### 2.34.1. Antiulcer Activity

*In Ayurvedic*. Locally, decoction of the leaves is employed in unhealthy ulcers and is an efficacious gargle for swollen gums and ulceration of the mouth [73].

*In Recent Studies*. The methanol leaf extract of* P. guyava* was administered at the doses of 500 and 1000 mg/kg orally, in rats for 10 days against ethanol induced gastric ulcer. The extract significantly decreases in ulcer indices compared to control [74].

*Active Constituents*. Quercetin, guaijaverin, flavonoids, and galactose-specific lecithins are considered.

### 2.35. *Rhus coriaria*

*Rhus coriaria* (*Anacardiaceae*) is commonly known as “sumach.” It is native to southern Europe. Chemical constituents in this plant are ellagic acid, gallic acid, isoquercitrin, myricitrin, and tannic acid [75].

#### 2.35.1. Antiulcer Activity

*In Ayurvedic*. It is generally used in the form of powder or extract; dose of the powder is 20 to 30 grains. Locally the paste mixed with charcoal powder is applied to unhealthy ulcers [75].

*In Recent Studies*. The hydro alcoholic extract of* R. coriaria* was administered at the doses of 145 and 248 mg/kg orally in rats against ethanol induced gastric ulcer. The extract significantly increases the healing of gastric ulcers [76].

*Active Constituents*. Flavonoids and tannins are considered.

### 2.36. *Sesbania grandiflora*

*Sesbania grandiflora* (*Fabaceae*) is commonly known as “basna.” It is locally called “akathi.” It is an ornamental plant and is found in the plains of Western Himalayas to Sri Lanka. Chemical constituents in this plant are saponins, tannins, and triterpenes [77].

#### 2.36.1. Antiulcer Activity

*In Folk Medicine*.* Sesbania grandiflora* leaves prepared in the form of soup and taken orally by the Valaiyan tribe of Alagarkoil Hills, Madurai district, Tamil Nadu, India, are used as vermifuge and against peptic ulcer. 50 mL of leaf decoction is taken orally on an empty stomach as vermifuge and against stomach ailments by tribal and rural people of Sirumalai Hills, Dindigul district, Tamil Nadu, India. Leaves are boiled in cow milk and orally taken in Kikuku village, Muleba district, Tanzania, for treatment of peptic ulcers. The boiled leaves are taken orally for ulcer by Paliyar tribals in Dindigul district of Tamil Nadu, India [77].

*In Recent Studies*. The ethanol leaf extract of* S. grandiflora* was administered at the dose of 400 mg/kg orally, in rats against aspirin, ethanol, and indomethacin induced gastric ulcer. The extract significantly inhibited gastric mucosal damage and reduced the basal gastric acid secretion [78].

*Active Constituents*. Tannins and saponins are considered.

### 2.37. *Shorea robusta*

*Shorea robusta* (*Dipterocarpaceae*) is commonly known as “sal tree.” It is locally called “taloora; kungiliyam.” It is common in the sub-Himalayan regions and the forests of Western Bengal. Chemical constituents in this plant are ursolic acid, tri and tetrehydroxy ursenoic acid, Asiatic acid alpha and beta amyrin, and mangiferonic acid uvaol [79].

#### 2.37.1. Antiulcer Activity

*In Ayurvedic*. Take* S. robusta:* 5, Cinnabar: 2, Mastiche: 3,* Calamus draco*: 3, and Ghee (10 parts). Mix and make an ointment; it is used for foetid ulcers [79].

*In Recent Studies*. The extract of* S. robusta* was administered at the doses of 150 and 300 mg/kg orally in rats against ethanol and pylorus ligation induced gastric ulcer. The extract significantly increases the gastroprotective activity as compared to control [80]. 

*Active Constituents*. Ursolic acid and amyrin are considered.

### 2.38. *Solanum nigrum*

*Solanum nigrum* (*Solanaceae*) is commonly known as “black nightshade berries.” It is locally called “manathakkali keerai.” It is cultivated throughout India. Chemical constituents in this plant are alkaloids, saponins, flavonoids, and phytosterols [81].

#### 2.38.1. Antiulcer Activity

*In Folk Medicine*. The fresh leaves are consumed for intestinal ulcer by Paliyar tribals in Dindugal district, Tamil Nadu, India [81].

*In Recent Studies*. Aqueous leaf extract of* Solanum nigrum* protected against pylorus ligation induced gastric ulcers in rats [82].

*Active Constituents*. Flavonoids are considered.

### 2.39. *Tamarindus indica*

*Tamarindus indica* (*Caesalpiniaceae*) is commonly known as “tamarind tree.” It is locally called “puli; puliyam-pazham.” This evergreen tree which is indigenous to South India is cultivated throughout India and Burma. Chemical constituents in this plant are pulp that contains tartaric acid 5%, citric acid 4%, malic and acetic acids, tartaric of potassium 8%, invert sugar 25–40%, gum, and pectin. Seeds contain albuminoids, fat, carbohydrates 63.22%, fibre, and ash containing phosphorus and nitrogen. Fruit contains traces of oxalic acid [83].

#### 2.39.1. Antiulcer Activity

*In Ayurvedic*. Decoction of the leaves is used as a wash for indolent ulcers and promotes healthy action [83].

*In Recent Studies*. The methanolic extract of the seed coat of* T. indica* at doses of 100 and 200 mg/kg significantly reduces the total volume of gastric juice and free and total acidity of gastric secretion in pylorus ligation induced ulcer model as compared to control [84].

*Active Constituents*. Tannins are considered.

### 2.40. *Terminalia chebula*

*Terminalia chebula* (*Combretaceae*) is commonly known as “myrobalan; Ink-nut; gullnut.” It is locally called “kaduk-kai.” This tree is wild in the forests of Northern India, Central Provinces, and Bengal and common in Madras, Mysore, and in the Southern parts of the Bombay Presidency. Chemical constituents in this plant are tannin (tannic acid) 45% and a large amount of gallic acid, lucilage, a brownish yellow colouring matter, and chebulinic acid which when heated in water splits up into tannic and gallic acids [85].

#### 2.40.1. Antiulcer Activity

*In Ayurvedic*. Ashes of Triphala mixed with sindhu salt (Saindhava, that is, Potassium Nitras or Nitricum) are dusted over syphilitic ulcers for washing away the exudation from the ulcers. Equal parts of dried myrobalans in combination with emblic and beleric myrobalans and catechu, both finely powdered and rubbed into a thick paste with sufficient ghee or some bland oil, make an excellent ointment as an application to aphthae for chronic ulcerations and ulcerated wounds [85].

*In Recent Studies*. Methanolic extract of* T. chebula* was administered in the doses of 250 and 500 mg/kg orally. Gastric lesion was induced by pylorus ligation induced ulcer and ethanol induced gastric ulcer. The extract showed significant reduction in gastric volume, free acidity, and ulcer index as compared to control [86]. 

*Active Constituents*. Tannins, gallic acid, chebulinic acid, and sorbitol are considered.

Some of the herbal drugs have been chemically characterized and the entities involved in the activity have been isolated. These are summarized in Table 1.

## 3. Conclusion

From this study we can conclude that studies with plant sources can result in novel and effective pattern of treatment. Current stalemates of modern medicine in the management of various ailments incline research tendencies to traditional medicine. In this respect, traditional medicine has introduced good protocols for treatment of various gastrointestinal disorders. All of the remedies presented here had adequate evidence from traditional or scientific source for their efficacy in management of ulcers.

According to the old hypothesis, acid secretion was thought to be the sole cause of ulcer formation and reduction in acid secretion was thought to be the major approach towards therapy. However, in the light of recent evidences this concept has changed. Now treatment of ulcer mainly targets the potentiation of the defensive system along with lowering of acid secretion.

Chemical substances derived from plants have been used to treat human diseases since the dawn of medicine. Roughly 50% of new chemical entities introduced during the past two decades are from natural products. Recent technological advances have renewed interest in natural products in drug discovery. Therefore, efforts should be directed towards isolation and characterization of the active principles and elucidation of the relationship between structure and activity. There are various medicinal plants and their extracts (containing active chemical constituents, e.g., tannins and flavonoids) that have significant antiulcer activity in in vivo experiments on animal models. Furthermore, detailed analysis of the active constituents of natural drugs should be directed towards clinical relevance. Standardization is indispensable to maintain reproducible quality in biological evaluation. Although the clinical efficacy of these preparations is reported by traditional practices, they have not been scientifically validated.

Ayurveda, the oldest medicinal system in the world, provides leads to find therapeutically useful compounds from plants. Therefore, ayurvedic knowledge supported by modern science is necessary to isolate, characterize, and standardize the active constituents from herbal sources for antiulcer activity. The combination of traditional and modern knowledge can produce better drugs for the treatment of peptic ulcer with fewer side effects.

It is apparent that experimental evaluation of herbal drugs for the treatment of gastric ulcer is rather impressive but very few have reached clinical trials and still few have been marketed. This shows that the benefits of research are not reaching the people to whom medical research is directed and hence the time, manpower, and resources are not efficiently utilized. Hence, pharmacologists need to take more active interest in evaluation of herbal drugs for potential antiulcer activity and standardization of such herbal drugs to be clinically effective and globally competitive.

## Figures and Tables

**Table 1:** Ulcer protective effect of some active constituents isolated from herbal drugs.